# Supplementary material for: TOSCCA: a framework for interpretation and testing of sparse canonical correlations
Source: Bioinform Adv. 2024 Feb 21;4(1):vbae021. doi: 10.1093/bioadv/vbae021 (PMC10919946; doi:10.1093/bioadv/vbae021)
Supplement: vbae021_Supplementary_Data [file vbae021_supplementary_data.pdf]

Supplementary material

A. Figures

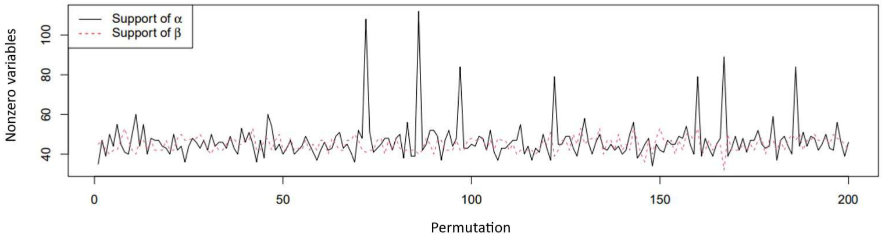

Fig. A1: Sparsity levels for the lasso and the fused lasso penalties over different permutations. For the PMA model, the same lasso (or fused lasso) penalty yields different number of nonzero variables.

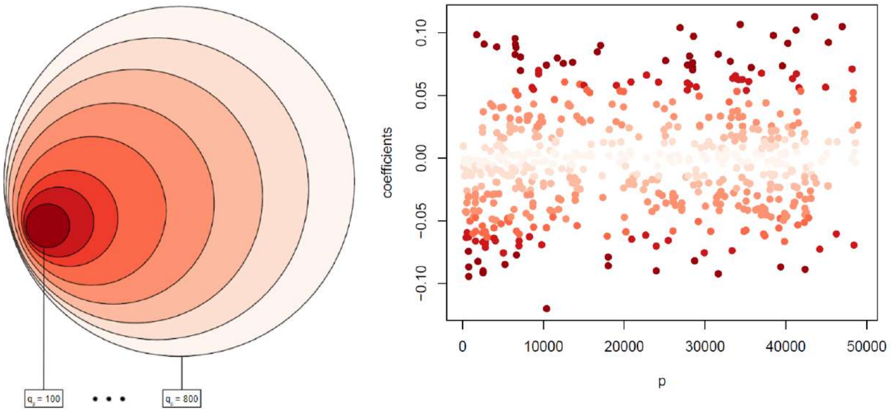

Fig. A2: Illustration of how sparser options, such as $q_\beta = 100$, are subsets of denser mode ones, say $q_\beta = 800$. That is, all the nonzero weights in $q_\beta = 100$, are included in $q_\beta = 800$ (left). Nonzero weights for 8 different choices of the threshold parameter ($q_\beta \in 100, 200, \ldots, 800$). We see that canonical weights sparser alternatives are included in denser choices (right).

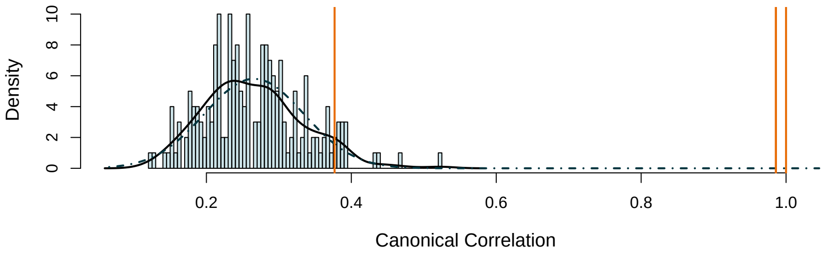

Fig. A3: Permutations for the simulations on Section **??**. The three true components are shown to be significant while the fourth (noise) is not.

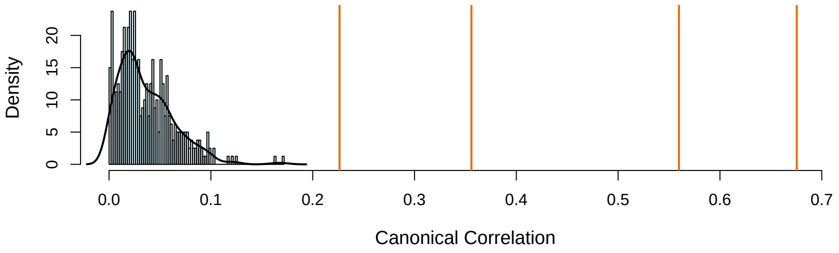

Fig. A4: Permutations for the GDCS data on Section **??**. Four components are shown to be significant.

B. Simulations

We generated the simulations according to the probabilistic interpretation of CCA by **?**. We assume there is some normally distributed latent process $\mathbf{z}$. where, again, K is the number of components.

$$\mathbf{Z} \sim \mathcal{N}(0, I_K)$$

The observed data is then simulated by conditioning on the latent variable $\mathbf{Z}$:

$$\boldsymbol{X}_1/\mathbf{Z} \sim \mathcal{N}(\boldsymbol{A}\mathbf{z} + \boldsymbol{\mu}_1, \Psi_1)$$

$$\boldsymbol{X}_2/\mathbf{Z} \sim \mathcal{N}(\boldsymbol{B}\mathbf{z} + \boldsymbol{\mu}_2, \Psi_2)$$

Here, $\boldsymbol{A}$ and $\boldsymbol{B}$ are matrices containing the K canonical vectors. Last, we assume $\boldsymbol{\mu}_1 = \boldsymbol{\mu}_2 = 0$. For our simulations we had $K = 3$, $N = 100$, $p = 2500$ and $q = 500$ and canonical weights of different sizes as displayed in Table A1.

Supp	$\boldsymbol{\alpha}$	$\boldsymbol{\beta}$
$k = 1$	10	10
$k = 2$	101	81
$k = 3$	21	51

Table A1. True support of canonical vectors

The false and true positive rates for each model are displayed in Table A2. The threshold parameters for TOSCCA were set to 100, from vastly bigger than some of the signal to just right for others. This choice will always impact the false positive rate. However, we observed in Figures **??** and A2, those weights which are *truly* zero tend to be distinctively pushed closer to zero.

K	**False positives rate**	**True positives rate**
$k = 1$	0.02	1
$k = 2$	0.0008	0.92
$k = 3$	0.004	1

Table A2. False and true positive rates for TOSCCA.

With these simulations we verify TOSCCA is able to recover the signal for each component.

C. Breast Cancer data

In this section we provide an analysis of the publicly available breast cancer data [?] used in **?** to evaluate PMA's performance on real data. This data has measurements on mRNA gene expression ($p = 19672$) and DNA copy number(q = 2149; array CGH data) for 89 patients.

Similar to **?**, we focus the analysis on matching the chromosomal location of the DNA variable and the location of the mRNA gene, in the these are often found close to each other. While in sparse canonical vectors. The reason for this is that these so-called cis-correlations are known to be very strong in these data, and they have a clear biological cause, because DNA codes for mRNA. Therefore, it is expected that these dominate the first components of the CCA. The authors of the PMA algorithm employed a semi-supervised approach by dividing the DNA measurements by chromosome (hence partly using estimate the canonical correlation for the 23 subsets, location information) and then performing separate CCAs for the 23 subsets.

We, however, believe CCA is, at its core, an explorative algorithm and therefore perform the analyses, both PMA and TOSCCA, unsupervised. That is, estimating the canonical correlations for the full data, for 23 components. We set the threshold parameters to 25 each for both DNA and the mRNA variables, yielding very sparse canonical vectors. In Figure A6 we show the chromosomal locations of the 25 nonzero weights for the canonical vectors estimated by TOSCCA (Figure A5a) and PMA (Figure A5b) for the first component. We also include subsequent components $k = 2, 3, 4$ in Figures A6a to A6c. Using this unsupervised analysis, we observe TOSCCA finds more matches between DNA and mRNA locations compared to PMA, in particular for components 1 and 4.

Additionally, we briefly discuss results for selection stability as it is a desired property of exploratory algorithms. We found both algorithms showed more variability in and canonical correlation. We found that both algorithms showed more variability in the estimation of canonical weights compared to the analysis in Section **??**. This is likely explained by the highly correlated data, which may have competing associations performing similarly. Therefore, different runs of the algorithms may select different nonzero weights for the first component. TOSCCA showed some improvement over PMA in selection stability for both dense and sparse choices of the penalties. PMA shows somewhat higher canonical correlations, due to it rendering less orthogonal results, thereby allowing overlap between subsequent components.

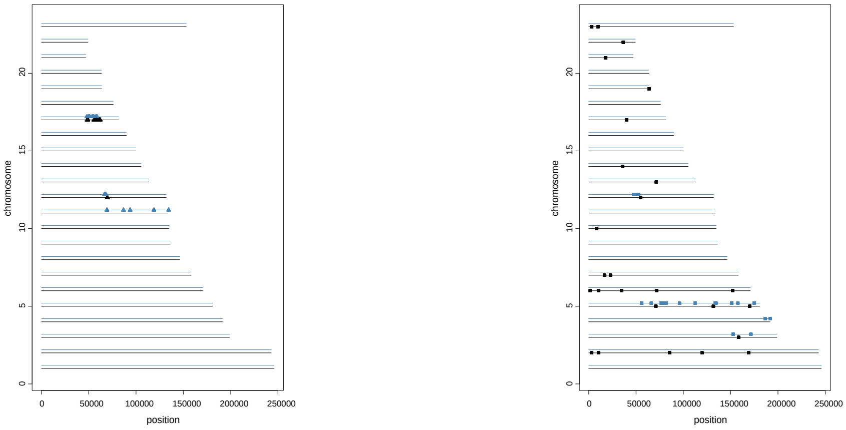

(a) TOSCCA (b) PMA

Fig. A5: Position of nonzero weights of the canonical vectors for component $k = 1$.

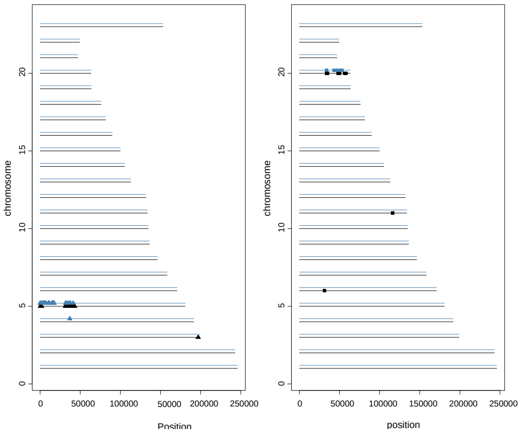

(a) k = 2

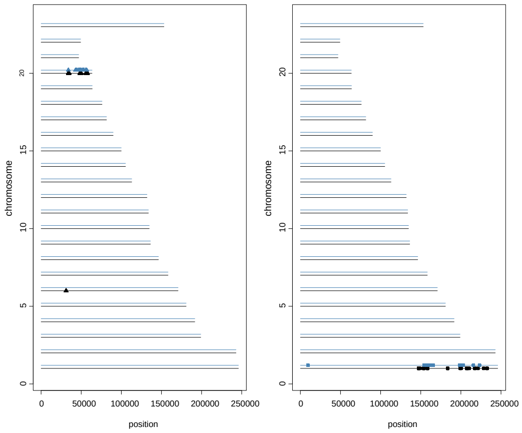

(b) k = 3

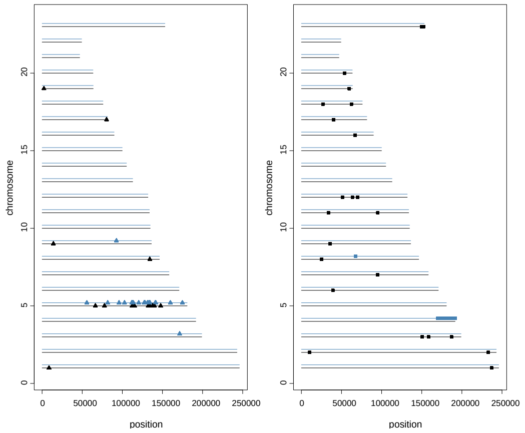

(c) k = 4, TOSCCA (left) and PMA (right)

Fig. A6: Position of nonzero weights of the canonical vectors for components $k \in 2, 3, 4$, TOSCCA (left) and PMA (right).

D. Sensitivity analysis

We carried out a sensitivity analysis for simulated data at 5 different signal-to-noise ratio (SNR). We defined SNR as the ratio of the signal variance over the noise variance. levels, SNR $\in 5, 2, 1, 0.5, 0.2$. We then compared TOSCCA's optimal sparsity levels to the true one, and evaluated the true and false positive rates. The simulations follow the same scheme as those described in section **??** of the main text and section B of the appendix. The canonical weights for this component depend on the size of p.The true coefficients in figures A7a and A10a are displayed prior to scaling the data. We evaluated TOSCCA's performance at sparsity levels ranging from 10 to 500 by a distance of 5. The true number of nonzeroes was 100 for the canonical vector $\boldsymbol{\alpha}$, and 80 for $\boldsymbol{\beta}$, while p, q and N remained as in section B. For brevity, we will only show results for $\boldsymbol{\alpha}$, which corresponds to the largest dataset.

Overall, the yielded correlations are similar across different sparsity levels as false positives are shrunk towards zero. The optimal number of nonzeroes tended to increase as the SNR decreases, which is expected as the true signal became masked by high levels of noise. However, the difference in the canonical correlation estimate was minimal once the signal had been included.

Figure A7 shows the true and estimated canonical vectors for all SNRs. The signal was correctly identified for all ratios but $SNR = 0.2$. This shows TOSCCA has the ability to recover the signal even in situations where there was twice as much noise as there was signal. The estimated weights were, of course, impacted by the proportion of noise in the data, and experienced more shrinkage the noisier the data was.

We then looked at signal recovery at $SNR = 1$ with $p_\alpha \in 50, 100, 500$. That is, sparsity levels that were smaller, equal and greater, respectively, of the true one. Figure A8 displays these results. For the first two sparsity levels, all the nonzeros belonged to the true signal. For the latter, the excess nonzero variables were visibly shrunk towards zero.

Figure A9 shows a grid of canonical correlation estimates for three different SNRs. For $SNR = 5$ (Figure A9a) and $SNR = 1$ (Figure A9b), where the signal is correctly discovered, the correlation estimates are very similar, especially once the signal has been included. It is therefore up to the researched to take into consideration their particular knowledge on the expected size of the associations and the interpretational constraints in choosing the sparsity levels.

We repeated the above analysis with now a $p = 100000$. We observed that the signal was correctly recovered for $SNR = 5$ and $SNR = 0.5$ (Figures A10b and A10e) with optimal sparsity level above true ones. Yet the false positives are distinctively shrunk towards zero. For $SNR = 2$ and $SNR = 1$ the optimal sparsity levels were both smaller than the true one. However, as previously mentioned, many sparsity levels performed similarly, as seen in Figure A12. It is up to the researcher to choose among what they believe is feasible and interpretable among the best performing sparsity levels. Then again, for $SNR = 1$ at the same three sparsity levels and observed that when the chosen sparsity level were smaller or equal to the true one, there are no false positives. Alternatively, when the chosen sparsity level was larger than the true one, the false positives were pushed towards zero.

Last, Figure A12 displays the grid search for the estimated correlations. We searched for sparsity levels from 10 to 460, spaced by 50. We chose to reduce the search as matrices $(\boldsymbol{A}_k, \boldsymbol{B}_k)$ becoming increasingly larger and take longer to compute (section **??**). The analysis of the grid search, nevertheless, remained the same as we observed a situation similar to the one described above. That is, estimates perform similarly once the signal has been detected.

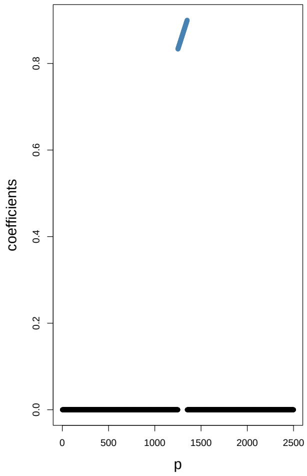

(a) True $\boldsymbol{\alpha}$

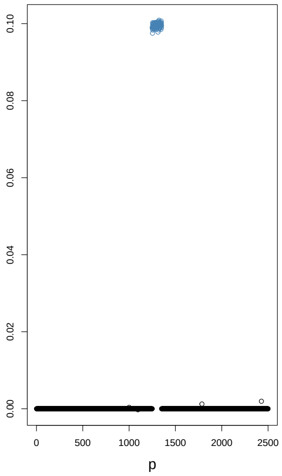

(b) Estimated $\boldsymbol{\alpha}$ with $SNR = 5$

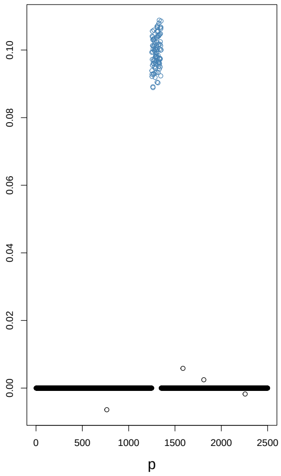

(c) Estimated $\boldsymbol{\alpha}$ with $SNR = 2$

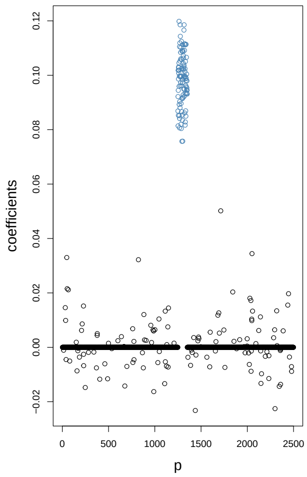

(d) Estimated $\boldsymbol{\alpha}$ with $SNR = 1$

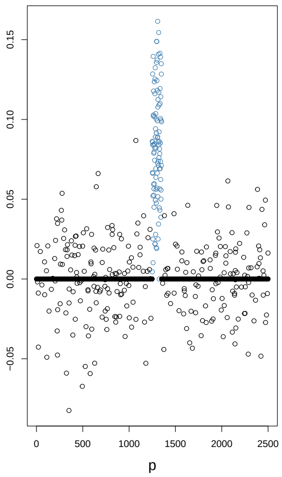

(e) Estimated $\boldsymbol{\alpha}$ with $SNR = 0.5$

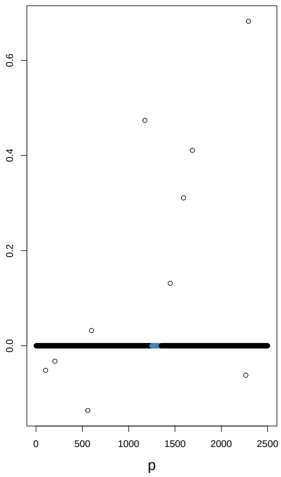

(f) Estimated $\boldsymbol{\alpha}$ with $SNR = 0.2$

Fig. A7: Signal recovery for different signal-to-noise ratio (SNR).

We have observed how TOSCCA performs for different signal-to-noise ratios and for two different dimensions of p. TOSCCA is able to recover the signal even when there is twice as more noise as there is signal, both for $p = 2500$ and $p = 100000$. The optimal sparsity values found with the available grid search show that many sparsity levels perform similarly once the true nonzero variables have been included in the canonical vectors. When the optimal sparsity level includes false positives, this are shrunk towards zero. These results conclude TOSCCA's ability to recover information in cases where both dimensionality and noise heavily interfere in the data exploration and signal discovery.

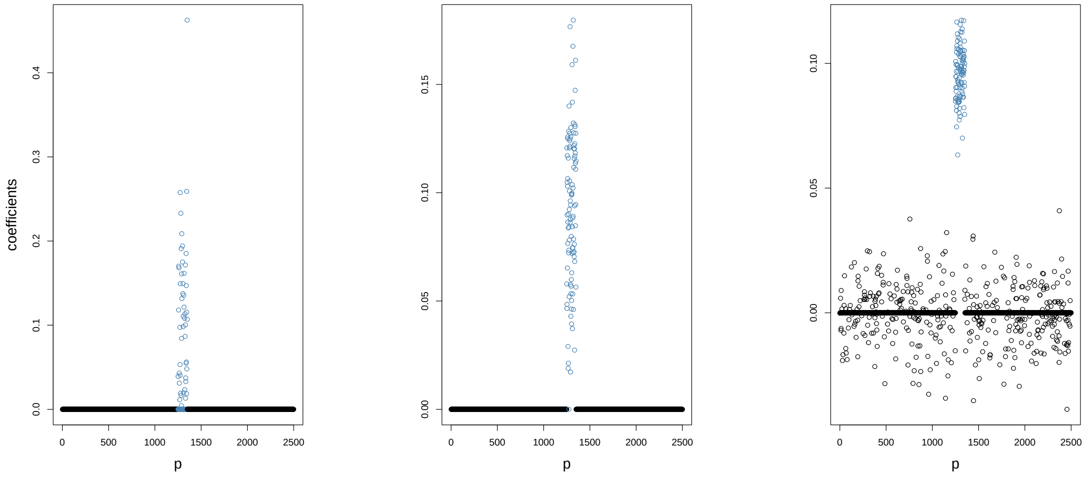

(a) $p_\alpha = 50$ (b) $p_\alpha = 100$ (c) $p_\alpha = 500$

Fig. A8: Signal recovery at different sparsity levels for $SNR = 1$.

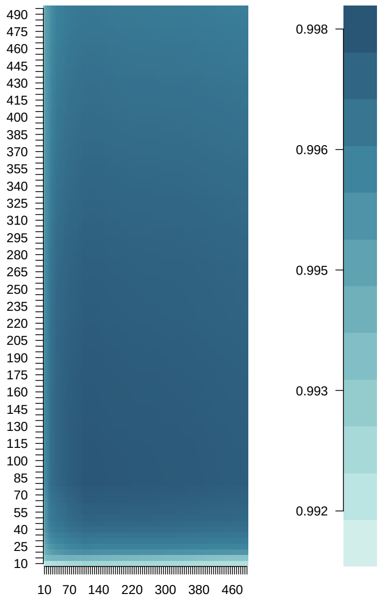

(a) $SNR = 5$

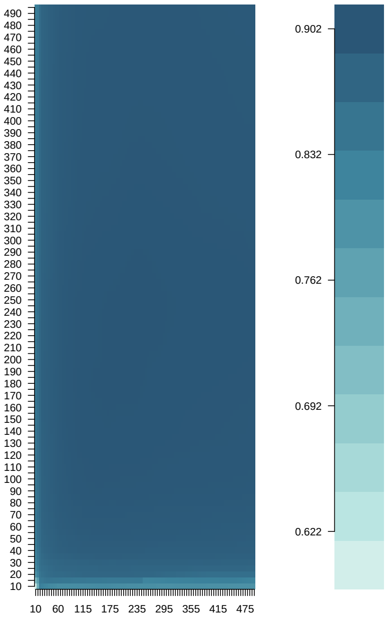

(b) $SNR = 1$

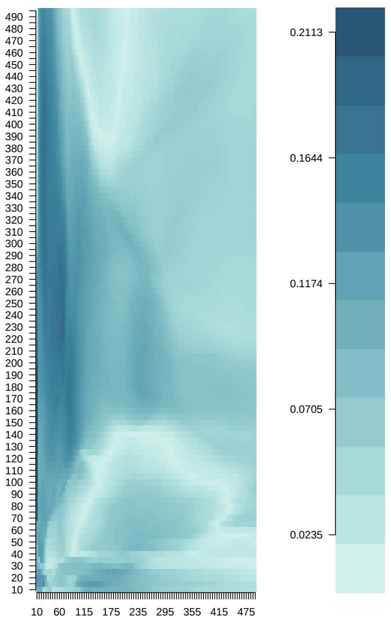

(c) $SNR = 0.2$

Fig. A9: Grid search for optimal sparsity level at different SNR.

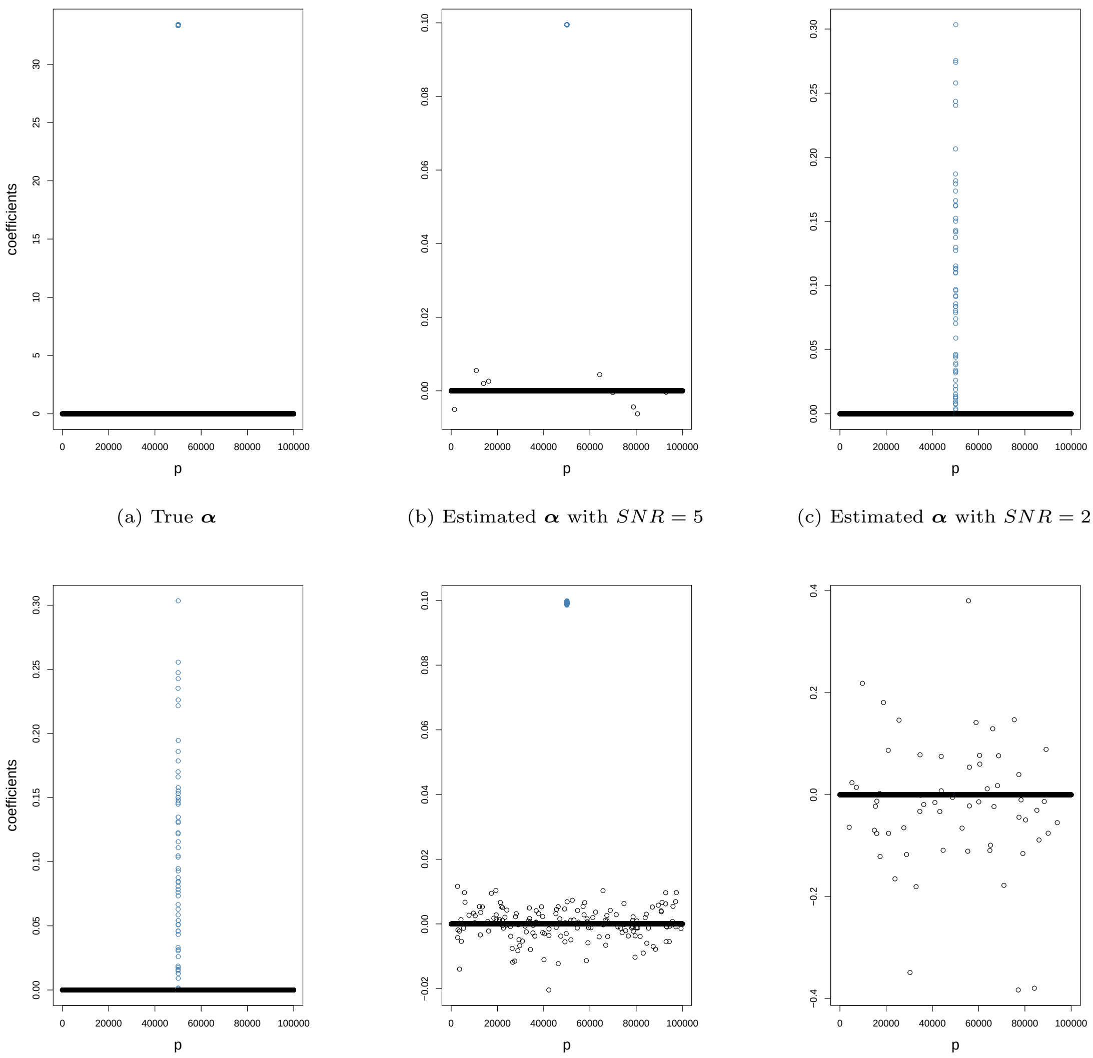

(a) True $\boldsymbol{\alpha}$ (b) Estimated $\boldsymbol{\alpha}$ with $SNR = 5$ (c) Estimated $\boldsymbol{\alpha}$ with $SNR = 2$

(d) Estimated $\boldsymbol{\alpha}$ with $SNR = 1$ (e) Estimated $\boldsymbol{\alpha}$ with $SNR = 0.5$ (f) Estimated $\boldsymbol{\alpha}$ with $SNR = 0.2$

Fig. A10: Signal recovery for different signal-to-noise ratio (SNR).

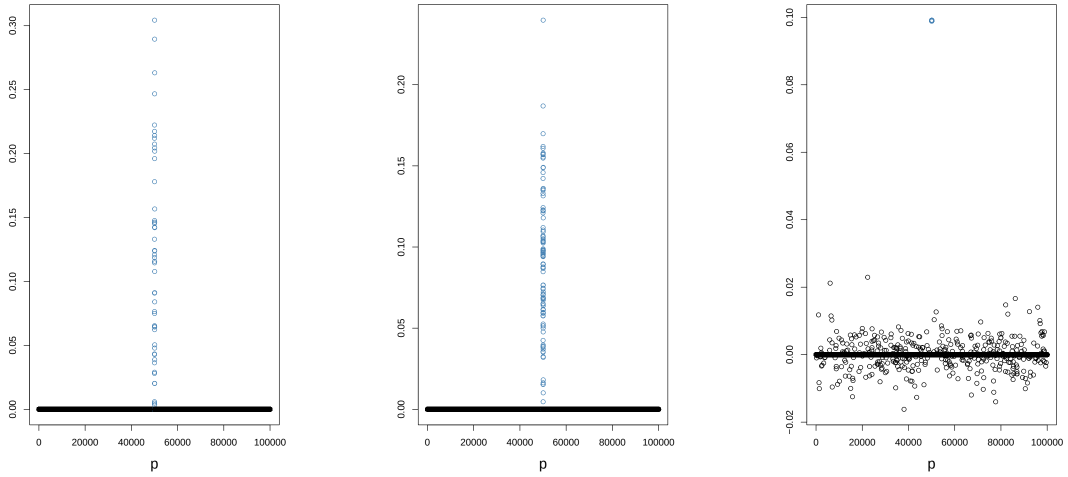

(a) $p_\alpha = 50$ (b) $p_\alpha = 100$ (c) $p_\alpha = 500$

Fig. A11: Signal recovery at different sparsity levels for $SNR = 1$.

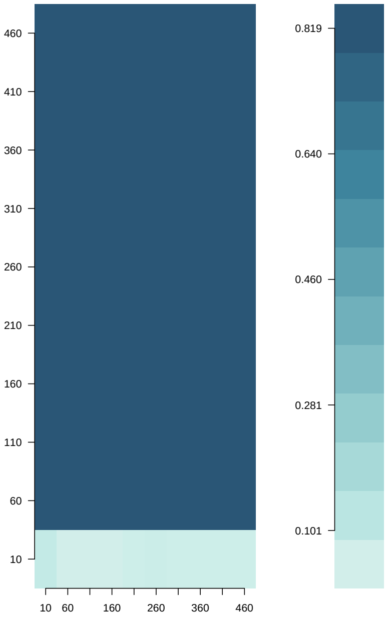

(a) $SNR = 5$

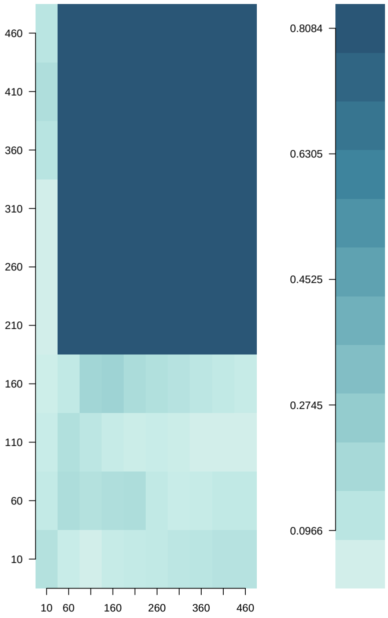

(b) $SNR = 1$

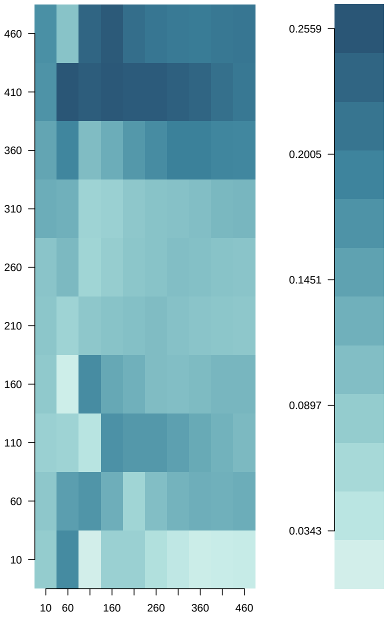

(c) $SNR = 0.2$

Fig. A12: Grid search for optimal sparsity level at different SNR.
